# The overestimation of concentric hypertrophy in patients with HFpEF as determined by 2D-echocardiography

**DOI:** 10.21542/gcsp.2022.11

**Published:** 2022-06-30

**Authors:** Mohammad F. Mathbout, Hussam Al Hennawi, Anwar Khedr, Gaurang N. Vaidya, Marcus Stoddard

**Affiliations:** 1 Medical University of South Carolina, Department of Cardiology, Charleston, South Carolina, USA; 2 Department of Internal Medicine, Jefferson Abington Hospital, Abington, PA, USA; 3 Department of Critical Care Medicine, Mayo Clinic Health System, Mankato, MN, USA; 4 University of Kentucky, Gill Heart Institute, Lexington, Kentucky, USA; 5 University of Louisville, Department of Cardiovascular Medicine, Louisville, Kentucky, USA

## Abstract

**Background:** Heart failure with preserved ejection fraction continues to pose multiple challenges in terms of accurate diagnosis, treatment, and associated morbidity. Accurate left ventricular (LV) mass calculation yields essential prognostic information relating to structural heart disease. Two-dimensional (2D) echocardiography-based calculations are solely limited to LV geometric assumptions of symmetry, whereas three-dimensional (3D) echocardiography could overcome these limitations. This study aims to compare the performance of 2D and 3D LV mass calculations.

**Methods:** A prospective review of echocardiography findings at the University of Louisville, Kentucky, was conducted and assessed. Normal ejection fraction (EF) was defined as >=52% in males and >=54% in females. The following calculations were performed: relative wall thickness (RWT) = 2x posterior wall thickness/LV internal diastolic dimension (LVIDd) and 2D LV mass = 0.8{1.04([LVIDd + IVSd +PWd]^3^ − LVIDd^3^)} + 0.6. Concentric hypertrophy was RWT >0.42 and LV mass >95 kg/m^2^ in females or >115 kg/m^2^ in males. The same cut-offs were used for 2D and 3D echocardiography.

**Results:** Echocardiographic findings for a total number of 154 patients in the study were investigated. There was a weak positive correlation between 2D and 3D LV mass indices (*R* = 0.534, *r*2 = 0.286, *p* = 0.001). Seventy patients had 3D EF >=45% with clinical heart failure (HFpEF). Among HFpEF patients, LV hypertrophy (LVH) was present in 74% of patients by 2D echocardiography and 30% by 3D echocardiography (McNemar test *p* = 0.001). Using 3D echocardiography as the reference, 68% of normal patients were misdiagnosed with LV hypertrophy by 2D echocardiography. Two-thirds of the patients with concentric remodeling by 3D echocardiography were misclassified as having concentric hypertrophy by 2D echocardiography (*p* = 0.001).

**Conclusion:** Adapting necropsy-proven LV mass index cutoffs, 2D over-diagnosed LV hypertrophy through overestimation of the mass, compared to 3D echocardiography. In turn, the majority of HFpEF patients showed no structural hypertrophy of the LV on 3D imaging. This suggests that the majority of patients with HFpEF may qualify for pharmacological prevention to prevent further progression to LV remodeling or LVH.

## Background

Among different parameters used to assess left ventricular (LV) function, left ventricular ejection fraction (LVEF) remains the most widely used echocardiographic parameter which provides an independent predictor of mortality and further direct patient management^1–3^. Operator-dependent 2-dimensional (2D) and 3-dimensional (3D) echocardiographic imaging provides both quantitative and qualitative assessments of the LV hemodynamic functions necessary for optimal cardiac evaluation. While both can assess LVEF, 2D echocardiography LVEF assessment is largely dependent on the reader’s experience and imaging plane with varying accuracy according to imaging quality^4^. Newly emerged techniques used for LV functional evaluation include temporal speckle-tracking echocardiography (STE), which depicts LV myocardial deformation parameters, including global longitudinal strain (GLS). Unlike EF evaluation, GLS provides reproducible readings subject to subtle changes in LV function prior to imminent changes in EF in different disease states amenable to medical treatment^5,6^. Moreover, different studies have reported more accurate mortality predictions associated with GLS than with EF^7–10^.

Although most outcome predicting studies have utilized 2D echocardiography (2DE) to evaluate LV function, 3D echocardiography (3DE) has been shown to provide superior LV size and function evaluations in terms of reproducibility and function^11^. This is largely due to overcoming apical foreshortening and the acquisition of measurements that are mainly based on direct volumetric measurements in the absence of geometrical presumptions. Of note, in the era of imaging advances, growing evidence has shown that 3DE has provides better visualization of LV morphology analysing different parameters including relative wall thickness (RWT) outlining early stages of myocardial hypertrophy confidently tied with further diagnostic and prognostic outcomes^12,13^. Therefore, we hypothesized that 3DE analytical tools can better predict ensuing myocardial changes in patients with HFpEF, making them candidates for an early course of pharmacological treatment.

### Methods

A prospective review of echocardiography findings at the University of Louisville, Kentucky, was conducted and assessed. Normal ejection fraction (EF) was defined as >=52% in males and >=54% in females. The following calculations were performed: relative wall thickness (RWT) = 2x posterior wall thickness/LV internal diastolic dimension (LVIDd) and 2D LV mass = 0.8{1.04([LVIDd + IVSd +PWd]^3^ −  LVIDd^3^)} + 0.6. Concentric hypertrophy was RWT >0.42 and LV mass >95 kg/m^2^ in females or >115 kg/m^2^ in males. Same cutoffs were used for 2D and 3D echocardiography.

## Results

**Figure 1.**
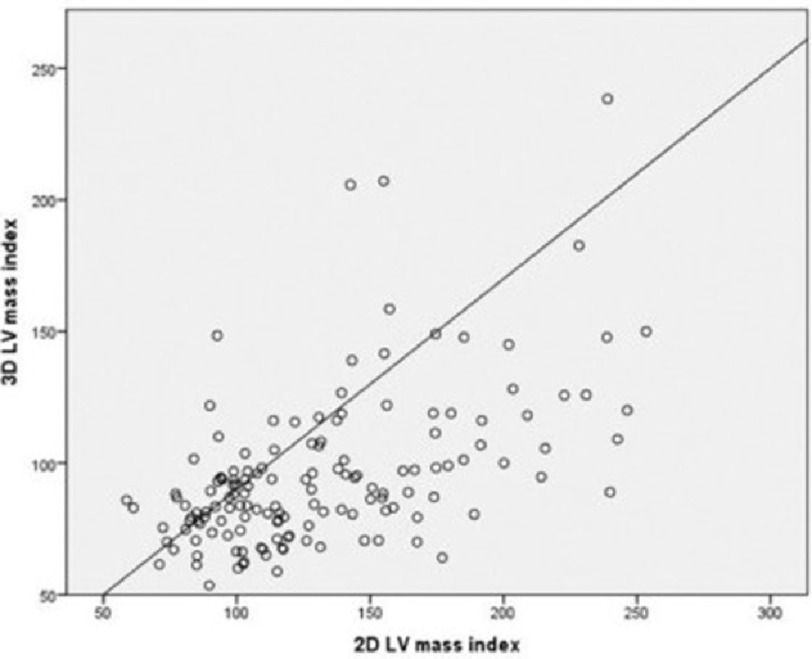
Comparison of 2D and 3D LV mass index.

Echocardiographic findings for a total number of 154 patients in the study were investigated. There was a weak positive correlation between the 2D and 3D LV mass indices (*R* = 0.534, r2= 0.286, *P* = 0.001) (Figure 1). Seventy patients had 3D EF >=45% with clinical heart failure (HFpEF). Among HFpEF patients, LV hypertrophy (LVH) was present in 74% of patients by 2D and 30% by 3D echocardiography (McNemar test *p* = 0.001). Using 3D echocardiography as the reference, 68% of the normal patients were misdiagnosed as LV hypertrophy by 2D (Table 1). Two-thirds of the patients with concentric remodeling by 3D echocardiography were misclassified as having concentric hypertrophy by 2D echocardiography (*p* = 0.001).

**Table 1:** Comparison of 2D and 3D interpretation of LV mass index.

	**2D interpretation**		**P-value**
	**Normal**	**LV Hypertrophy**	
**3D interpretation**	**Normal**	16 (32%)	33 (68%)	0.04
	**LV Hypertrophy**	2 (10%)	19 (90%)	

## Discussion

The pathogenesis of HFpEF is still not completely understood. The pathophysiology of HFpEF can be explained by two theories. The old conventional theory holds that systemic hypertension is the primary cause of left ventricular remodeling. Concentric left ventricular hypertrophy, fibrosis, and diastolic dysfunction result from pressure overload, leading to left atrial hypertension and remodeling. These processes can lead to pulmonary hypertension and atrial fibrillation. Finally, diastolic dysfunction in the left ventricle causes right ventricular and atrial remodeling, as well as concomitant right ventricular diastolic and systolic dysfunction^14,15^. In addition to systemic hypertension, a novel emerging theory considers metabolic disorders such as obesity, metabolic syndrome, and type 2 diabetes mellitus. These pro-inflammatory comorbidities produce microvascular endothelial inflammation. This results in coronary microvascular inflammation, decreased density, cardiac interstitial fibrosis, and increased oxidative stress, leading to cardiomyocyte hypertrophy and stiffness. The previous factors result in myocardial hypertrophy, remodeling, and dysfunction^14,16,17^.

Left ventricular wall remodeling is considered one of the most important pathophysiological factors predisposing to overt heart failure. Different patterns of left ventricular (LV) remodeling have been described, including normal geometry, concentric remodeling, concentric hypertrophy, and eccentric hypertrophy. Stemming from geometrical changes that can be detected at earlier stages, the current consensus on LV chamber measurements advises the characterization of LV geometry based on echocardiographically determined LV mass index (LVMI) and relative wall thickness (RWT)^18^. Healthy geometry is identified by normal LVMI and RWT. Compared to other disorders, concentric remodeling is characterized by increased RWT, whereas both eccentric and concentric hypertrophy are marked by increased LVMI with normal RWT in the former and increased RWT in the latter. Moreover, concentric hypertrophy can be differentiated by an increase in short-axis diameter compared to an increase in myocyte length in eccentric hypertrophy, which is evident microscopically^19^. Although LV geometric abnormalities can be detected at early stages of LV remodeling, heart failure has over the years been staged by LV ejection fraction (LVEF) instead of LV geometry. This has recently conflicted with experts’ opinions recommending that early recognition of geometric abnormalities can preserve normal heart function and provide accurate estimation beyond what is expected from ejection fraction alone^20^.

Left ventricular remodeling and hypertrophy were found to be the most common abnormal geometric abnormalities in patients with HFpEF in most epidemiological studies, registries, and clinical trials (Tables 2, 3)^21–27^. In addition, patients with HFpEF are reported to have more prominent concentric hypertrophy than those with hypertensive heart disease who do not have HFpEF^12,28^. It was also found that LVH had a reverse correlation with exercise capacity. Among all geometric types, patients with LV concentric hypertrophy showed the greatest exercise limitation due to reduced contractility and chronotropic incompetence^29^. Moreover, LVH was found to be a strong predictor of heart failure hospitalization, cardiovascular death, or aborted cardiac arrest. In this study, LV concentric remodeling and hypertrophy were the most common abnormal geometric findings associated with an increased risk of hospitalizations inferred from the TOPCAT trial^27^.

**Table 2:** Prevalence of LV concentric remodeling and hypertrophy in patients with HFpEF in selected epidemiological studies and registries.

	Normal geometry	Left ventricular remodeling	Left ventricular concentric hypertrophy	Left ventricular eccentric hypertrophy
Olmsted County^5^	31%	27%	26%	16%
ARIC study^6^	5%	20%	73%	2%
Northwestern registry^7^	12%	28%	48%	12%

**Table 3:** Prevalence of LV concentric remodeling and hypertrophy in patients with HFpEF in selected epidemiological studies and registries.

	Normal geometry	Left Ventricular remodeling	Left ventricular concentric hypertrophy	Left ventricular eccentric hypertrophy
PARAMOUNT^8^	72%	14%	7%	7%
I-PRESERVE^9^	46%	25%	29%	0%
PARAGON-HF^10^	46%	33%	12%	9%
TOPCAT^11^	14%	34%	43%	9%

Heart failure with preserved ejection fraction (HFpEF) is a clinical syndrome that accounts for half of all heart failure (HF) patients and has been increasing in prevalence attributing to major cardiovascular mortality^30^. HFpEF expands beyond abnormalities strictly to LV diastolic function and is considered a spectrum of diseases that encompasses limitations in cardiac, vascular, and peripheral functions^30^. On the other hand, LV diastolic dysfunction plays a cardinal role in the pathophysiology of HFpEF^31^. LV diastolic dysfunction is characterized by an impairment of heart muscle relaxation, an increase in viscoelastic chamber stiffness (decreased compliance), or a combination of the two^32,33^. This results in symptomatic HF stemming from the congestion of the vascular system and other vital organs, giving rise to a range of symptoms, including dyspnea, impairment of daily activities at rest, and exertion.

Hemodynamic impairments associated with circulatory pump failure predispose patients to recurrent hospitalization, diminished quality of life (QoL), and decreased survival. Early HFpEF phenotyping is crucial to halt further progression and may impart necessary measures for targeted therapies to this specific subpopulation of patients positioned to attain the greatest benefit^31^. Cardiovascular imaging, and echocardiography in particular, plays a vital role in the diagnosis and assessment of HFpEF, which evaluates cardiac structure, hemodynamics, and function^34^. While the diagnosis of HFpEF is clear in symptomatic patients with signs of overt congestion, diagnosing euvolemic patients with marked exertional dyspnea poses a considerable challenge^35–37^.

### Conclusion

HFpEF continues to pose multiple challenges concerning accurate diagnosis, treatment, and associated morbidity. Cardiovascular imaging provides important information necessary for accurate diagnosis at early stages in patients who may require treatment to halt further progression. In particular, echocardiography assesses cardiac function and accurately identifies abnormal geometric changes; therefore, clinical suspicion warrants evaluation.

Since LV remodeling and concentric hypertrophy are the most common geometric changes in HFpEF and constitute the cornerstone of HFpEF pathogenesis, it is essential to identify these changes before progression to more advanced stages. This study adds value to the diagnostic and prognostic utility of 3D echocardiographic functional indices to identify LV remodeling and concentric hypertrophy early in order to risk-stratify patients and drive their management accordingly.
